# Epigenetics as an Evolutionary Tool for Centromere Flexibility

**DOI:** 10.3390/genes11070809

**Published:** 2020-07-16

**Authors:** Laura Leo, Marcella Marchetti, Simona Giunta, Laura Fanti

**Affiliations:** 1 Istituto Pasteur Italia, Dipartimento di Biologia e Biotecnologie “Charles Darwin”, “Sapienza” University of Rome, 00185 Rome, Italy; laura.leo@uniroma1.it (L.L.); marcella.marchetti@uniroma1.it (M.M.); simona.giunta@cantab.net (S.G.); 2 Laboratory of Chromosome and Cell Biology, The Rockefeller University, New York, NY 10065, USA

**Keywords:** centromere, neocentromere, holocentromere, CENP-A, repetitive sequences, centromere evolution

## Abstract

Centromeres are the complex structures responsible for the proper segregation of chromosomes during cell division. Structural or functional alterations of the centromere cause aneuploidies and other chromosomal aberrations that can induce cell death with consequences on health and survival of the organism as a whole. Because of their essential function in the cell, centromeres have evolved high flexibility and mechanisms of tolerance to preserve their function following stress, whether it is originating from within or outside the cell. Here, we review the main epigenetic mechanisms of centromeres’ adaptability to preserve their functional stability, with particular reference to neocentromeres and holocentromeres. The centromere position can shift in response to altered chromosome structures, but how and why neocentromeres appear in a given chromosome region are still open questions. Models of neocentromere formation developed during the last few years will be hereby discussed. Moreover, we will discuss the evolutionary significance of diffuse centromeres (holocentromeres) in organisms such as nematodes. Despite the differences in DNA sequences, protein composition and centromere size, all of these diverse centromere structures promote efficient chromosome segregation, balancing genome stability and adaptability, and ensuring faithful genome inheritance at each cellular generation.

## 1. Introduction

Centromeres are specialized chromatin regions that establish the assembly site for the kinetochore, a complex protein structure that mediates the attachment of spindle microtubules to chromosomes, thus permitting proper chromosome segregation during cell division. In all organisms studied thus far, it has been shown that no DNA sequence is either necessary or sufficient for centromere identity. The only known exception is *Saccharomyces cerevisiae*, whose centromeres are specified by a conserved 125-bp sequence (reviewed in [1]). Instead, centromeres are defined by the deposition of the histone H3 variant centromeric protein A (CENP-A) that replaces canonical histone H3 in centromeric nucleosomes [2,3,4]. CENP-A is regarded as the main epigenetic component of eukaryotes’ centromere, yet some organisms lack this centromeric histone variant. CenH3 was lost in at least four lineages of holocentric insects [5]. Besides insects, it is known that in kinetoplastids, a group of unicellular flagellated eukaryotes, not one CENP-A homolog has been identified [6,7] and, in contrast to holocentric insects, they possess an unconventional kinetochore [8].

CENP-A chromatin underlies the formation of the constitutive centromere-associated network (CCAN) [9,10,11,12,13], and in mitosis, serves as a template for assembly of the kinetochore to enable the chromosome for the correct segregation [14]. 

CENP-A is recruited at different stages of the cell cycle depending on the organism but, unlike canonical histones, its loading is uncoupled from DNA replication [15,16]. In human cells, CENP-A deposition occurs in late telophase or early G1 [17]. In *Drosophila*, Cid (homolog of CENP-A) is incorporated at different times depending on developmental stage and on the specific cellular culture, but it is generally also found to be loaded in late mitosis/early G1 [18,19,20,21,22]. In *S. pombe* on the other hand, CENP-A homolog is incorporated during G2 [23]. This process is extensively regulated by preloading complexes [24,25,26,27] (reviewed in [28]) containing accessory factors; specific chaperons for CENP-A, for instance in human cells identified as HJURP [29,30] and in *Drosophila* as Cal1 [20,31,32]; and cell cycle-dependent phosphoregulation (reviewed in [33]). The specification of a narrow time window for CENP-A loading onto chromatin and why it needs to be decoupled from the replication-dependent assembly of canonical histones remain open questions. 

CENP-A undergoes a variety of post-translational modifications (PTMs) including acetylation, methylation, phosphorylation and ubiquitylation (reviewed in [34,35]). In particular, ubiquitylation of CENP-A at lysine 124 has been proposed as an epigenetic marker of the centromere location. According to the proposed octamer model, two dimers of the nucleosome are distributed between the two centromere DNA strands during replication. The ubiquitylated old CENP-A is recognized by HJURP that favors a new ubiquitylated CENP-A deposition in a heterodimerization-dependent manner. This allows centromere spatial positioning and epigenetic inheritance [36,37] (reviewed in [38]). 

Although centromere DNA sequences are not conserved between species, and in some cases not even between centromeres of the same species, they generally contain DNA rich in repeated sequences, in particular tandem satellite DNA such as human alpha-satellite that can extend for mega bases, or SATIII as seen in *Drosophila* and in humans. In mice, two types of repetitive DNA sequences are associated with centromeres: major satellite repeats that are located in the pericentromeric heterochromatin and the minor satellite repeats located in the centric chromatin (reviewed in [39,40]). 

Recent works have shown the centromeric presence of mobile elements, specifically retrotransposons, in several species including *Drosophila*, [41,42], humans [43] and maize [44], probably contribute to the establishment and maintenance of eukaryotic centromeres while promoting their variability (reviewed in [45,46]). 

## 2. Centromere Flexibility in Response to Stress

Because of their essential function in the cell, centromeres may have evolved high flexibility and mechanisms of tolerance to preserve their functionality following stress originating from within or outside the cell. Indeed, substantial changes in centromere integrity and overall size can cause chromosome aneuploidy, segregation and structural defects (reviewed in [47]) that can induce cell death with consequences on health and survival of the organism as a whole.

DNA damage to the centromere may have multiple origins (reviewed in [47]). First of all, centromeres are subjected to mechanical stress during anaphase due to the microtubules that pull them towards the poles. Moreover, it has been proposed that alterations of the mitotic spindle are a possible cause of segregation and structural defects. In addition, spindle defects, that expose chromosomes to excessive forces, can generate centromeric double-strand breaks (DSBs), possibly leading to carcinogenesis [48]. It was shown that lagging chromosome formation is linked to the accumulation of DNA damage markers, such as γH2AX, MDC1 and 53BP1, and activation of the ATM/Chk2 response [49]. Defects in DNA replication is another possible cause of stress for centromeres (reviewed in [50]). Because of their repetitive nature, the centromeric chromatin forms complex secondary structures [51,52], representing a problem during replication and inducing a stalled fork. This could make this region prone to replication errors and recombination events that disrupt the integrity or structure of the centromere, causing aneuploidy (reviewed in [53]). 

Since the presence of repeated sequences at the centromere is a characteristic conserved during evolution, it has been suggested that the centromeric sequences were selected for the capacity to preserve the functionality of the centromere even following changes in the DNA sequence, providing a favorable environment for centromere maintenance and stability through formation of particular three-dimensional structures [54]. To date, the level of tolerance for sequence changes, repeat content and copy numbers remains unclear. However, centromeric-repeated sequences remain a necessary feature for the building of human artificial chromosomes that can be inherited through cell division. Only direct seeding of CENP-A can bypass this sequence requirement [55]. 

Analysis of extended chromatin fibers has shown that blocks of CENP-A containing nucleosomes are interspersed with H3 containing nucleosomes in both *Drosophila* and humans [16,56]. It has been proposed that these nucleosome blocks form a cylindrical three-dimensional structure in which H3 containing nucleosomes are mainly oriented inwards and CENP-A containing nucleosomes outside where they contact with the kinetochore proteins [57]. This three-dimensional structure could make the centromeric function more effective, and have a role in the stability of the centromere itself. 

The centromere also responds to stimuli that reach the cell from the external environment. Any perturbing agent that changes the cellular microenvironment can be considered a source of stress and potentially harmful to the centromere’s essential function. Stressing factors can be both abiotic, such as heat, cold, UV light, heavy metals etc. [58,59,60], and biotic, such as parasites and infectious agents. Physiological changes derived from development and differentiation are also underlined by profound epigenetic and transcriptional transitions that contribute to diverse forms of stress for the cell [61]. Stressors that directly challenge the integrity of the genome by generating DNA damage or perturbing the DNA replication process can also impinge on centromeres. Notably, centromere DNA instability has also been associated with cancer and cellular senescence [62]. 

In the last years, several studies on different organisms have shown that heat shock induces transcriptional activation of centromeric and pericentromeric regions [63,64,65,66,67] (reviewed in [68,69]). Stress-dependent non-coding-RNA expression has been detected in human cells. Mainly, they are transcribed from satellite III (Sat III) repeats, located in the pericentromeric region of chromosome 9. This activation depends on the activity of heat shock factor 1 (HSF1) that binds to the Sat III sequence and drives the production of long non-coding Sat III RNAs [70,71]. A large number of stressing factors other than heat shock induce both Sat III RNAs and the formation of nuclear stress bodies (nSBs) [58,59]. Under heat shock, HSF1 recruits acetyltransferases such as GCN5, TIP60 and p300/CBP to pericentric heterochromatin and the consequent targeted hyperacetylation in turn directs the recruitment of proteins required for Sat III transcription by RNAP II [72]. Transcriptional activation at pericentric heterochromatin is thought to occur through the replacement of H3K9 methylation with H3K9 acetylation [72], but the molecular mechanisms involved are still poorly characterized. Furthermore, since the core repetitive alpha-satellite at the human centromere is largely devoid of H3K9me3, but instead shows a chromatin state associated with poised transcription, it is unclear how transcriptional activation differentially affects pericentromeres from centromeres, especially since transcription start sites are poorly mapped within these repetitive regions. Nevertheless, both centromeric and pericentromeric transcripts, with or without induction by external agents, have been implicated in various cellular functions, such as the transmission of epigenetic information, differentiation, and the cellular defense to stress [58,70,71,73] (reviewed in [68]). Furthermore, the disruption of transcription, as in the upregulation of centromeric lncRNAs, is associated with cancer, suggesting that transcriptional control must be maintained in both regions.

It is also widely assumed that transcription is a process closely related to the centromeric function in several organisms, including fission yeast [74,75], humans [39,76], and *Drosophila* [77,78], and that it is particularly associated with CENP-A deposition [79,80,81,82]. It has been proposed that the passage of RNA Pol II along the centromeric sequences creates an epigenetic environment which favors the deposition of CENP-A [83]. Indeed, the active form of RNA pol II localizes to centromeres during mitosis in mammals [39,84]. In *Drosophila* cells, the activity of RNA polymerase II temporally coincides with de novo deposition of CENP-A [78] (reviewed in [85]). These findings support the idea that transcription is coupled to CENP-A loading and that it is required for CENP-A deposition into centromeric chromatin. Studies in *Drosophila* show also that the destruction of the centromeric transcripts affects centromere stability, suggesting that not only the transcription process but also the transcription products have a role in centromere maintenance and function [76,77]. Indeed, a layer of RNAs constitutes a structural element of the mitotic centromere–kinetochore interface, as observed by electron microscopy [86]. 

In addition to repetitive satellite sequences, transposable elements (TEs) are abundant components of (peri)centromeric heterochromatin, as shown in humans [87,88] and *Drosophila* [89,90]. TEs and Sat are structurally related from an evolutionary point of view (reviewed in [91]) and are still largely biologically active [92,93] (reviewed in [94]). Centromeric TEs, in addition to satellite sequences, have also been shown to be transcribed [42,80,95,96] (reviewed in [97]). Some models have been proposed where retrotransposons could produce non-coding RNAs with a role in the centromere specification [43,98]. In fact, retroelements could contribute to induce breaks under specific circumstances and thereby increase the number of repeated sequences through retrotransposition and recombination events, maintaining the correct centromere size [99,100] (reviewed in [101]). 

There is evidence that suggests that the expression of centromeric and pericentromeric repeats is epigenetically regulated, involving changes of DNA methylation and histone modifications (reviewed in [68]). In general, the establishment and maintenance of centromeric chromatin are also epigenetically regulated [102] (reviewed in [103,104]). An increasing number of proteins responsible for establishing and maintaining active or silent chromatin expression have been identified. Among them are proteins involved in histone modifications, chromatin remodeling, meCpG-maintenance or binding. Several of these proteins have been found in (peri)centromeric heterochromatin through a detailed analysis of their distribution on metaphase chromosomes at both human and mouse centromeres. While several of them are heterochromatin-associated proteins binding both centromeric and pericentromeric regions, a few others are exclusively kinetochore-associated proteins, such as Sin3A, PCAF, MYST and BAF180 [105].

**Figure 1:**
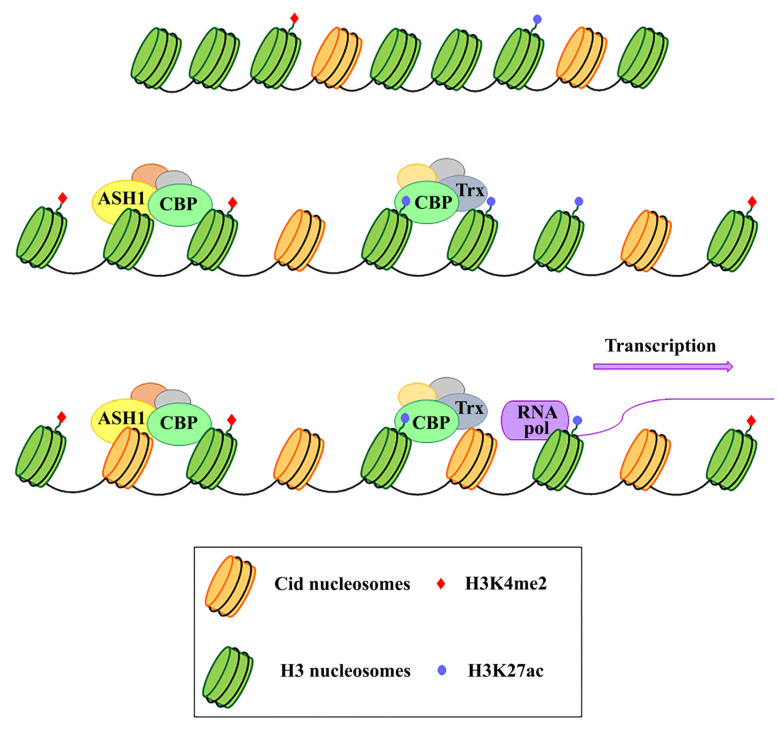
Schematic representation of the functional role of Ash1, CBP, and Trx proteins in the centromeric region. These proteins work by modifying the epigenetic state of the centromeric chromatin. In particular, on H3 histones, Ash1 dimethylates lysine 4 (H3K4me2) and CBP acetylates lysine 27 (H3K27ac) within the centromeric region. Trx works by inducing chromatin opening which, in turn, favours both CENP-A/Cid deposition and activation of transcription.

In *Drosophila*, but also in human cells, the analysis on immunostained chromatin fibers has shown a clear presence of H3K4me2 and H3K36me, typically associated with active chromatin in Cid (CENP-A in *Drosophila*) labeled centromeric chromatin, and of H3K9me3, a repressive histone marker in adjacent heterochromatin [106,107]. Instead, no centromere-specific accumulation of these specific markers has been found at the chicken centromere [108] and plant centromere [109]. Recent studies have demonstrated that transcriptional activators of euchromatic genes belonging to the trx-G group, in particular Trithorax (Trx), Ash1 and CBP, co-localize with Cid-containing chromatin [110]. Ash1 and CBP depletion through post-transcriptional silencing of the respective coding genes causes a decrease in Cid at the centromere and a significant increase in chromosomal aberrations at all phases of mitosis, such as decondensation, lagging chromosomes and the generation of chromosomal fragments. Instead, Trx depletion causes the same chromosomal aberrations without affecting the overall level of Cid protein. Immunofluorescence analysis using antibodies against H3 histone has shown that Trx functions open up the chromatin, making it accessible to transcription factors. In fact, Trx depletion induces a compaction of the centromeric chromatin with a higher concentration of H3-containing nucleosomes. Ash1 and CBP are transcriptional activators which work through histone modifications. In particular, Ash1 methylates H3K4me2 and CBP acetylates H3K27ac at the centromere. Both modifications are specific for active chromatin and their decrease is related to a depletion of Cid [110]. It has been proposed that a balance between methylation and acetylation could create an epigenetic environment that favors Cid deposition [111]. Alternatively, it could be hypothesized that the activating epigenetic modifications have the function of preserving a euchromatic region inside the heterochromatic domain necessary for CENP-A/Cid loading (Figure 1). Whichever the mechanism, the failure to open the centromeric chromatin is incompatible with Cid deposition. It is not known whether the euchromatic epigenetic environment directly favors the Cid deposition through recruitment of Cal1 chaperone or whether it favors the transcription of centromeric sequences such as Sat III or centromeric transposons, which in turn are required for Cid deposition. Disentangling these two roles would be an important issue to address. 

It has been shown that Sat III in both *Drosophila* and mammals, and centromeric transposons, are weakly transcribed [42,58], but it is not clear what their role in centromeric function could be. There is also the possibility that centromeres’ basal transcription is not causative but a mere consequence of the chromatin opening for Cid/CENP-A loading, without holding being necessary for centromeric functionality. However, given several studies have shown that the direct inhibition of centromeric RNAs causes overall reduction in CENP-A at centromeres [76,77,80,84,112,113,114,115,116], the alternative possibility would imply that transcription may be a consequence of chromatin opening, while transcription products play a direct role in CENP-A deposition and centromeric stability. 

In addition to the regulation of transcription as described above—derived from epigenetic changes, rapid evolution of DNA sequences and/or proteins—centromere flexibility can also manifest in other ways. The centromere position can also shift in response to an altered chromosome structure, such as through the generation of a neocentromere, a new chromosomal locus for CENP-A seeding outside of the endogenous and physiological locus, deprived of repetitive sequences [117,118,119,120,121]. Furthermore, centromeric proteins and the kinetochore complex can change also in response to fluctuations in kinetochore protein levels (reviewed in [122]). Some organisms, such as *Caenorhabditis elegans*, have diffuse centromeres (holocentromeres) of unclear evolutionary advantage [123]. Despite the differences in DNA sequences, proteins composition and centromere size, all of these diverse centromere structures promote efficient chromosome segregation, balancing genome stability and adaptability, and ensuring faithful genome inheritance at each cellular generation.

## 3. Neocentromere as a Functional and Evolutionary Model for Centromere Biology

**Figure 2:**
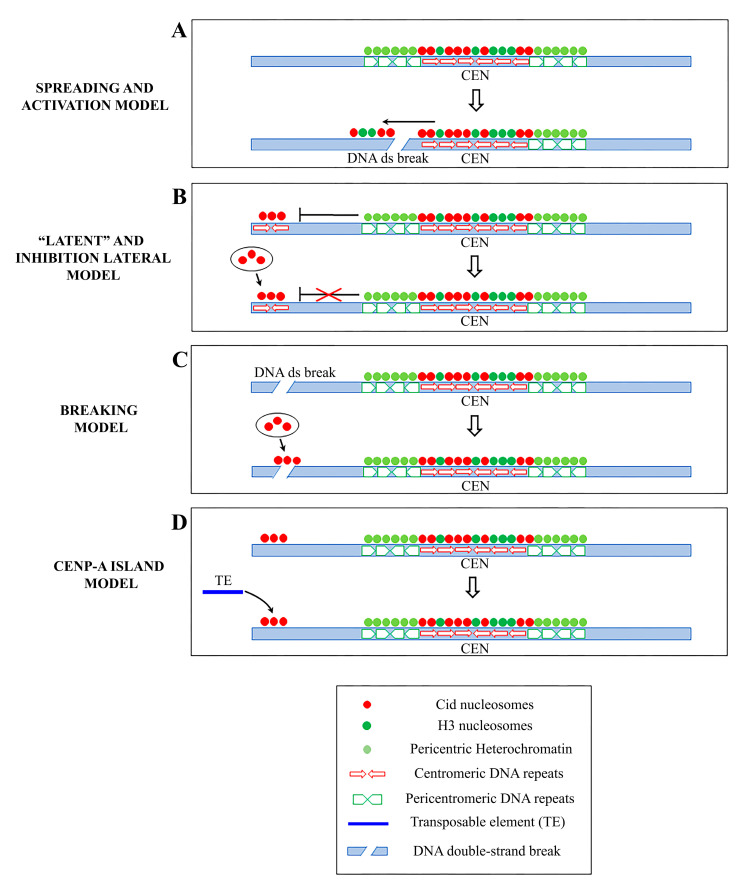
Schematic representation of the principal models of neocentromere formation: (**A**) Spreading model and activation model: centromeric markers spread from centromere into adjacent areas inducing neocentromere formation near endogenous centromeres following a DNA double-strand break (DSB) (**B**) Latent centromere model and inhibition lateral model: ectopic sites along a chromosome have an intrinsic ability to perform centromeric activity, but they are repressed by a dominant centromere. When the endogenous centromere is inactivated, one of these sites becomes active and functionally competent. (**C**) DNA breaking model: a neocentromere emerges at a breaking site where centromeric protein A (CENP-A) is rapidly recruited. (**D**) CENP-A island model: CENP-A is deposited on islands of nucleosomes scattered along the chromosomes. A random insertion of a transposable element (TE) in a CENP-A-ectopic site represents the first step towards the formation of a new, fully functional centromere.

How and why neocentromeres appear in a given chromosome region are intriguing questions. It has been proposed that de novo centromeres might represent different scenarios: “latent” centromeres [124,125,126], which are locations of ancestral centromeres following centromere repositioning events [127], (reviewed in [128]), or euchromatic regions, where centromeric markers have spread from adjacent areas, inducing neocentromere formation near endogenous centromeres (Figure 2A). In the chicken, CENP-A is also found in pericentromeric regions and it is able to trigger neocentromere formation when the endogenous centromere is damaged [108]. Neocentromeres have also been produced experimentally in *Drosophila*. In a study, a subtelomeric chromosome fragment containing a functional neocentromere was isolated after the irradiation of a minichromosome derived from the *Drosophila X* chromosome [118]. In other work, an identical segment of DNA was released from various sites, but neocentromere was formed only from cuts immediately adjacent to the centromeric chromatin [129]. These results support the model that formation of the neocentromere may depend on the proximity to an endogenous centromere.

However, some observations suggest that neocentromeres are often formed in distal chromosomal regions, usually separated from endogenous centromeres by long tracts of DNA, and they cannot be explained as formed by the spread of centromeric activity in *cis*. For example, in the *bw^D^ Drosophila* model, the heterochromatic region that acquires neocentromeric activity is located at the opposite end of the chromosome arm from the original centromere [119]. In these cases, the spread of centromeric activity in *cis* is unlikely to happen. However, the centromeric epigenetic marks may be able to spread in *trans* through an incorrect pairing of a non-centromeric-repeated DNA tract with an endogenous centromere [129]. These findings underscore the centromere’s essential role and the necessity to have a “back up” in case centromeres suddenly become dysfunctional.

A different model for neocentromere formation called the “lateral inhibition model” (Figure 2B) [130] supports the existence of many sites along a chromosome that are potentially able to perform centromeric activity, but that are normally repressed by a dominant centromere. In fact, just like absence of a centromere (acentric), multiple centromeres are also dangerous to chromosome stability. Dicentric chromosomes are subjected to mitotic bridges, chromosome breakage, and aneuploidy. According to this model, neocentromeres might be expected to form whenever the endogenous centromeres are inactivated or deleted (reviewed in [130]). However, the mechanisms of activation and/or repression of de novo centromere formation remain unclear.

It has been shown that a neocentromere is capable of forming near telomeres at the end of the chromosome [131]. This substantiates the hypothesis formulated by Agudo et al. [132] (reviewed in [133]) that the centromere evolved from telomere during the evolutionary origin of the eukaryotic chromosome. According to the “Centromere From Telomere Hypothesis” (CFTH), the accumulation of mobile elements at the breaking site produced by the linearization of ancestral circular DNA molecule generated the proto-telomere. The progressive expansion of these repeated elements caused the sub-telomeric sequences to be recognized as a new cargo by tubulin-based cytoskeleton, which evolved in a proto-centromere. The ensuing genomic instability, which also led to chromosomal fusions, seems to promote the centromere complexity and the evolution of metacentric chromosomes (reviewed in [134]). Support to this hypothesis is also found in the discovery that the telomeric transposons *Het-A* and *Tart* of *Drosophila* are localized in the centromeric heterochromatin of the Y chromosome [132]. This implies that telomeres and centromeres can share types of sequences, epigenetic factors and structural characteristics, allowing a functional interchange between them (reviewed in [134]). Human neocentromeres lack alpha-satellite DNA, but they were found to be indistinguishable in terms of protein content, except for the absence of CENP-B. This is expected due to CENP-B binding specifically to a motif named the CENP-B box [135], which is exclusively found within a subset of alpha-satellite monomers. Notably, gene-knockout experiments in mice have shown that CENP-B is not essential for centromere function during mitosis and meiosis [136,137,138]. This is corroborated by the absence of CENP-B box in the human *Y* chromosome, where centromere specification and function entirely rely on CENP-A and associated factors [139]. 

Studies in primates show that relocation of the centromere within a chromosome may occur via neocentromere formation. The X chromosomes of three primate species share an identical order of genetic markers, but different centromeric locations [140]. The absence of rearrangements on these chromosomes suggests that centromere repositioning occurred due to the appearance of a new centromere and not by translocation of an existing centromere.

Recently, Palladino et al. [131] using the lacI-lacO system, has shown that targeting Cal1 to chromosomal regions outside the original centromere induces the deposition of Cid and the formation of a new centromere at different locations, even at large genomic distance from the endogenous site. 

Zeitlin et al. [141] speculated that a neocentromere could emerge at site of breaks (Figure 2C), following the observation that CENP-A is rapidly recruited to DNA double-strand breaks. This point of view is also supported by Ventura et al. [127], who noticed the closeness of the breakpoint to the neocentromere location in some studies. 

It has been shown that CENP-A is produced in excess of the needs for centromeres and that it is deposited around the original centromere and on islands of nucleosomes scattered along the chromosomes [42,108,142], only to be cleared during replication [42,143]. It is possible that some of these islands accumulate a quantity of CENP-A that predisposes them to acquire a potential centromeric function in case the endogenous centromere is damaged. In this case, not all chromosomal regions are equally and simultaneously predisposed to acquire a centromeric activity. 

A heterochromatic environment seems favorable to neocentromere formation, as was shown in several organisms such as fission yeast [144] and *Drosophila* [119,145], especially given the key role of heterochromatin in forming a boundary to contain centromere expansion. On the other hand, several new ectopic neocentromeres appear to emerge in gene-poor regions, but in the absence of satellite DNA. However, in any new site where a centromere is formed, the selective pressure induces the accumulation of repetitive DNA until a heterochromatic environment is restored to halt the spread. Neocentromeres may also arise at euchromatic loci [108], but selective pressure probably acts to disadvantage these neocentromeres in order to avoid interfering with the gene transcription. The importance of the heterochromatic environment is also shown for those centromeres that naturally lack repeated sequences. As demonstrated in chicken DT40 cell lines by a 4C-seq analysis, non-repetitive centromeres and neocentromeres are transiently associated with heterochromatin in a three-dimensional arrangement during interphase [146]. Furthermore, despite lacking long tracts of tandemly repeated sequences, human neocentromeres are shown to be associated with known heterochromatic proteins [147,148]. 

Another mode of neocentromere formation may involve transposable elements (TE). Notably, stress induces the activation of TE, causing their transposition to new locations [67,149,150,151,152]. It is then possible that following chromosomal insult or rearrangements, a single TE may be sufficient to initiate CENP-A recruitment via its nascent transcription, breaks generation and other specific chromatin alterations associated with the “jump” and/or the TE reintegration into a new locus [98]. However, we favor an alternative hypothesis, that a random insertion of a TE per se does not trigger neocentromere formation unless it hits a CENP-A ectopic site. The concomitance of preexisting ectopic CENP-A and transposition may represent the first step towards the formation of a new centromere (Figure 2D). Accordingly, in *Drosophila S2* cells, we noticed islands of Cid throughout the chromosome fibers (as first observed in [110]) indicating that ectopic CENP-A clusters may be sites of potential neocentromeres. The subsequent CENP-A ubiquitylation could allow the neocentromere to be inherited during cell divisions [38]. Over time, the accumulation of other repetitive sequences through new transpositions or duplications of existing sequences in these CENP-A ectopic “hot spots” would create a genetic and epigenetic landscape for the evolution of a complex centromere that can functionally replace the endogenous one.

## 4. Holocentromere

Nematodes, some insects and species of plants belonging to the flowering plants have diffused centromeres throughout their chromosomes, and these are described as “holocentric”. From a cytological point of view, holocentric chromosomes do not present a primary constriction in metaphase—a hallmark narrowing found in stereotypical metacentric or sub-metacentric chromosomes—and do not have a designated place along the chromosome for spindle microtubule attachment. Instead, the whole chromosomal surface is bound and segregated. Furthermore, the sister chromatids must migrate in parallel to the spindle poles during mitotic anaphase, and have inherent problems in meiosis because spindles can attach to bivalents in a random fashion. Interestingly, several solutions have evolved to allow accurate meiotic segregation of holocentric chromosomes (reviewed in [153]). 

For most species, a scattered polycentric centromere arrangement is reflected in cenH3 dynamics during the cell cycle. During interphase, cenH3 is found dispersed, while in prophase it moves to form a pattern of small foci along chromosomes, and during metaphase it becomes a composite linear axial line along each sister chromatid [4,154,155]. Genomic evidence suggests that many holocentric chromosomes lack tandem repeats and have cenH3 binding sites distributed over a wide variety of unique sequences throughout the chromosome, as expected. Furthermore, recent identification of holocentric insects that lack CENH3 demonstrates that the centromere-specific histone marker can be dispensable while retaining canonical kinetochore components [5]. 

The nematode *C. elegans* is the exemplar case of a holocentric organism. Recently, the chromosomal localization of histone cenH3 was determined in *C. elegans* by ChIP–chip analysis [156]. It was found that ~50% of the genome can be associated with cenH3, showing complete absence of particular DNA sequences to control cenH3 incorporation. Importantly, the distribution of cenH3-containing regions was inversely correlated with genes transcribed in the germline and within the early embryo when the pattern of cenH3 incorporation was established [156]. This suggests that transcription in this case excludes, instead of promoting, cenH3 incorporation, underscoring a different modality of incorporation and maintenance for cenH3 in this species. These features may have allowed karyotypes to change without compromising holocentric meiosis [157]. 

**Figure 3:**
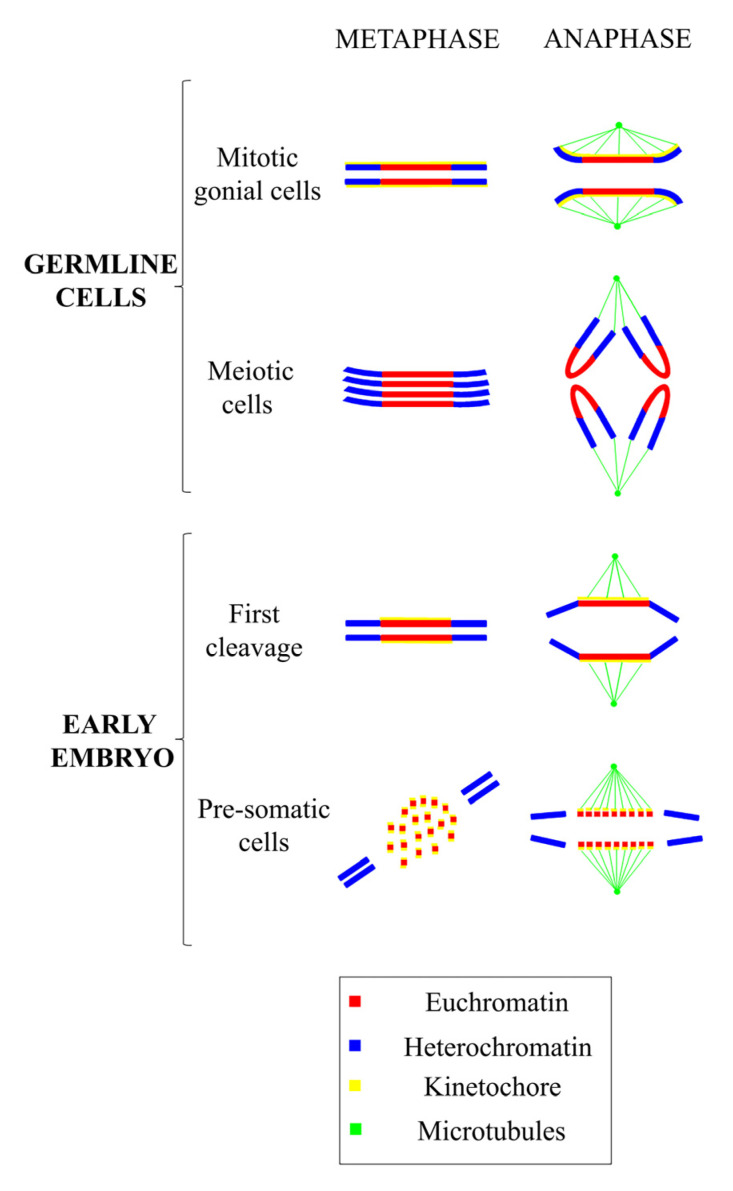
Diagrammatic representation of the variations in the centromere position and chromatin and kinetic activity in different cell types. Note, kinetic activity is retained exclusively by euchromatin in embryonic presomatic cells, in gonial cells by the entire chromosome and in meiotic cells only by telomeric heterochromatin.

Fundamental studies on the regulation of diffuse centromeres have been carried out on the nematode *Parascaris equorum*. In this organism, as in *C. elegans*, development is strictly mosaic, and each cell performs a predetermined number of cell divisions before differentiation. A consequence of this developmental system is that an induced death of a single cell can be lethal to the embryo because it cannot be replaced at the embryonic stage. Furthermore, in *Parascaris*, there is the phenomenon of chromatin diminution in somatic cells, which does not occur in germ cells. In embryonic somatic cells, both terminal and intercalary heterochromatin with no detectable kinetochore activity is eliminated by fragmentation, producing about 60 small chromosomes that are equipped with centromeres and that segregate correctly during mitosis [158]. In gonial cells, heterochromatin is not eliminated, and both heterochromatin and euchromatin retain kinetochore activity. Finally, in meiotic cells, centromeric activity is restricted to the terminal heterochromatic regions to which the spindle microtubules attach in the absence of a kinetochore plate. Therefore, in *Parascaris*, there is both structural and regional variability in relation to the cell type. In the embryo’s somatic cells, the centromeric activity is restricted to euchromatin, while in gonial cells it is diffused over different chromatin environments, and in meiotic cells is restricted to telomeric heterochromatin (Figure 3). Therefore, *Parascaris* and other nematodes represent interesting models to study epigenetic centromere organization for the presence of three different centromere states under physiological conditions. 

## 5. Conclusions

Proper segregation of chromosomes during cell division is essential for the survival of the cell and the whole organism. Therefore the centromere, a complex structure used for this function, has adapted during evolution to respond to changes in the cellular microenvironment as well to those in the external environment. The conflict between the need for functional stability and variability dictated by the environmental changes has been resolved with a wide flexibility through various epigenetic mechanisms. In order to keep the correct centromere functionality, epigenetic mechanisms can buffer possible centromere structural variations. Alternatively, such mechanisms can induce the formation of a neocentromere in the case of disruption of the old one. To this regard, several studies on the centromeres of diverse animal and plant organisms have shown that the centromeric flexibility affects both the structure and location of the centromere. Monocentromeres are located in a specific region of the chromosome, but if necessary, for example following inactivation of the endogenous centromere, neocentromeres can emerge in other sites with different sequences and protein content. 

Some organisms, such as nematodes, evolved diffuse/continuous centromeres along the chromosome. Depending on the developmental needs, centromeres modify their size so that only some regions have the ability to bind microtubules, with or without kinetochores. Moreover, holocentric chromosomes also have radically different patterns of kinetochore proteins compared to monocentric chromosomes [159]. 

By definition, holocentromeres occupy many DNA sequences (reviewed in [104]). In addition, for regional centromeres, although they have a preferential location within gene-poor region environments, there is a wide array of sequence types that retain capabilities to perform their function. Notably, neocentromeres emerge in gene-free regions and subsequently accumulate repeated sequences to restore a suitable environment, implying that in spite of centromere sequence flexibility and diversity seen throughout evolution, repetitive and gene-poor loci retain preferential characteristics for centromere formation and function.

Holocentromeres are the most striking example that the flexibility of the centromere is the adaptive result of the evolution of organism development and physiology. Nematodes such as *Parascaris* lose the terminal and intercalary heterochromatin in all presomatic blastomeres during early embryogenesis [158]. While the maintenance of heterochromatin is necessary for the germ line quality, it seems unnecessary in somatic cells where it may be an expensive burden. Moreover, it has been shown that some germ-line specific genes that are eliminated together with heterochromatin from somatic cells exist [160]. It was also proposed that position effect following the chromatin elimination could influence the gene expression (reviewed in [161]). The consequence of this chromatin diminution is the fragmentation of euchromatin into numerous and small chromosomes that segregate correctly since they have diffused centromeres. However, this opens the question of how heterochromatin can be lost if it has kinetic activity. The answer is via cell-specific centromeric activity, as described above. A further element of plasticity is given by the kinetochore morphology to enable chromosome segregation. In *Parascaris*, the kinetic activity in meiotic cells is restricted to heterochromatic terminal regions that interact directly with spindle fibers without kinetochore plates [162]. Instead, in somatic cells, a ladder-like kinetochore structure mediates the association of spindle fibers with centromere [163]. This and other examples suggest that centromere flexibility is also reflected in kinetochore rapid adaptation with the aim of continuously ensuring faithful chromosome segregation under widely different circumstances.

Altogether, holocentric organisms represent fascinating systems of a different epigenetic specification of centromeres from regional and point centromeres that may hold important aspects of chromosome segregation that are applicable across all species.
